# Research progress on the mechanism of hyperuricemic nephropathy based on multi-omics technique: A review

**DOI:** 10.1097/MD.0000000000040975

**Published:** 2024-12-20

**Authors:** Kaiqing Li, Xue Xia, Tong Fu, Yanchun Ma, Yingwei Wang, Mingming Fan, Songyan Wang, Guoli Xing, Ying Tong

**Affiliations:** a Heilongjiang University of Chinese Medicine, Harbin, China; b Brandeis University, Waltham, China.; c The First Affiliated Hospital of Heilongjiang University of Chinese Medicine, Harbin, China

**Keywords:** hyperuricemia, hyperuricemic nephropathy, intestinal flora, metabolomics, multi-omics technology, transcriptomics

## Abstract

Hyperuricemic nephropathy is a metabolic disease in which renal uric acid deposition and excretion are impaired due to elevated levels of uric acid in the blood, leading to impaired renal tubule function and chronic renal disease. Hyperuricemic nephropathy is one of the important complications of hyperuricemia, which seriously affects the quality of life and prognosis of patients. The pathogenesis of hyperuricemic nephropathy involves a variety of factors, including: amino acid metabolism disorder, energy metabolism abnormality, increased nucleotide metabolism, lipid metabolism disorder and bile acid metabolism imbalance, REDOX process disorder, cell cycle and apoptosis imbalance, signal transduction and inflammatory response enhancement, and intestinal flora imbalance. In recent years, omics techniques such as metabolomics, transcriptomics and intestinal microecology have been used to reveal the metabolic, gene and microflora characteristics of hyperuricemic nephropathy from different levels, as well as their interactions and regulatory mechanisms. This paper reviews these studies, analyzes the existing problems and challenges, and puts forward future research directions and suggestions, aiming at providing new theoretical basis and practical guidance for the prevention and treatment of hyperuricemic nephropathy.

## 1. Introduction

**Figure 1.**
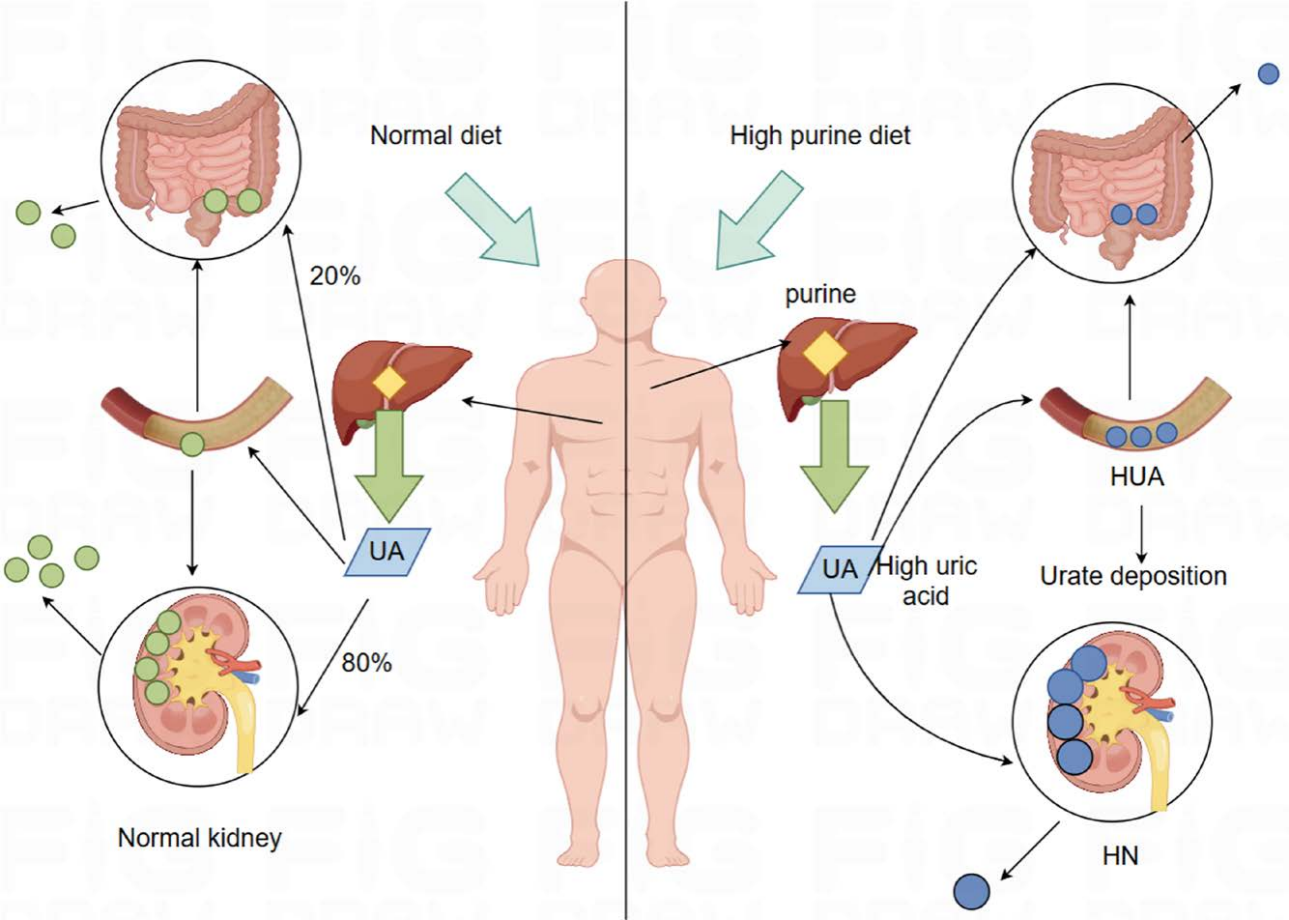
Normal UA metabolic pathway and UA metabolic imbalance resulting in HUA induced kidney damage. HUA = hyperuricemia, UA = uric acid.

Hyperuricemia nephropathy (HN) is a chronic metabolic disease in which uric acid (UA) crystals are deposited in the kidney and cause kidney inflammation, fibrosis and necrosis due to Hyperuricemia (HUA).^[1]^ HUA is a chronic metabolic disease resulting in increased UA concentration in the blood due to purine metabolism disorder or reduced UA excretion.^[2]^ Purines are substances that exist in the human body and are mainly involved in energy supply and metabolic regulation.^[3]^ UA is the final product of purine oxidation.^[4]^ Under normal circumstances, UA can be excreted from the body through the kidneys and intestines to maintain the balance of UA in the blood.^[5]^ However, due to poor living habits or a variety of factors leading to the decline of kidney function, UA metabolism will be unbalanced, and the concentration of UA in the blood will then increase.^[6]^ As shown in Figure 1. In recent years, with the development of economy and the change of lifestyle, the prevalence of HUA and HN has gradually increased,^[7–9]^ which has a serious impact on the health and quality of life of patients.^[10,11]^ HUA and HN are not only closely related to gout, but also to a variety of chronic diseases, such as chronic kidney disease,^[12]^ hypertension, diabetes, cardiovascular disease, etc., which increases the death risk of patients. At present, common methods for the treatment of HN include vegan diet,^[13]^ reducing blood UA level,^[14]^ increasing urine volume,^[15]^ alkalinizing urine,^[16]^ dissolving or discharging UA stones,^[17]^ etc. However, these methods often cannot fundamentally solve the problem and may cause other complications. Therefore, exploring the pathogenesis of HN is the key to improve the therapeutic effect and prevent recurrence. Scholars in China and other countries have made a lot of useful exploration on this, and believe that the regulatory mechanism of treatment of HN is effective through multi-component, multi-target and multi-link integration. However, because HN is a complex chronic disease caused by multiple factors, it is often limited to elucidate the pathogenesis of HN from a single pharmacodynamic level. Therefore, it is necessary to understand the pathogenesis of HN from a holistic perspective. With the rapid development of life science and the advent of the era of big data, multi-omics technologies such as metabolomics, transcriptomics and intestinal flora have emerged. The exploration of metabolites, transcriptomes and environmental microbial diversity provides a powerful means for in-depth research on the pathogenesis of HN. In this paper, we will review the recent progress in exploring the mechanism of HN based on single omics or multi-omics techniques, and look forward to the future research directions and challenges, in order to provide new theoretical basis and practical guidance for the prevention and treatment of HN.

## 2. Discussion on the mechanism of single omics technique in treating HN

### 2.1. Study on the mechanism of metabolomics in HN

Metabolomics is a high-throughput analysis technology to determine the small molecule compounds produced or consumed in the metabolic process of organisms, which can reflect the physiological and pathological state of organisms. At present, metabolomics has been widely used in the study of HUA and its related complications, including gout,^[18]^ kidney disease,^[19]^ and cardiovascular disease.^[20]^ Metabolomics enables analysis of different HN sample types and experimental models to reveal their unique metabolic characteristics and mechanisms of action. Metabolomics analysis found that several metabolic pathways such as amino acid metabolism,^[21]^ lipid metabolism,^[22]^ energy metabolism,^[23]^ purine metabolism,^[24]^ and glycerophospholipid metabolism^[12]^ are closely related to kidney injury, indicating that the influence of uric acid on the kidney is not limited to direct crystallization deposition, but also involves the regulation and interference of other metabolic factors. Zhang et al^[25]^ used Ultra-performance liquid chromatography–mass spectrometry (UPLC-MS) to detect plasma and fecal metabolite levels and metabolic pathways in HUA rats. Abnormal levels of metabolites such as bile acids, acylcarnitine and sulfocholylglycine were found, as well as disorders of metabolic pathways such as lipid metabolism, lipid signaling, hormone synthesis, unsaturated fatty acid absorption and tryptophan metabolism. Li et al^[26]^ used non-targeted metabolomics analysis of liquid chromatography-mass spectrometry (LC-MS) to establish an animal model of HUA by knockout the urate oxidase (Uox) gene capable of decomposing UA in mice. Through comparative analysis of metabolites in kidney and plasma of normal mice and Uox-Ko mice, the results showed that the renal pathway disorders of purine metabolism, amino acid biosynthesis, tryptophan metabolism and neuroactive ligand-receptor interaction in Uox-Ko mice were correlated with renal function, and the decrease of betaine in plasma and the increase of biotin were associated with renal function. Wei X et al^[27]^ discussed the changes in serum metabolic characteristics of HUA patients with hyperlipidemia by using the metabolomics method of UHPLC-MS. The results showed that the metabolic pathways of alanine, aspartate, and glutamate were significantly reduced in these patients, while the biosynthesis of linoleic acid, phenylalanine, tyrosine, tryptophan, and the metabolic pathways of glycine, serine, and threonine were significantly increased. These changes of metabolic pathways may reflect the pathophysiological processes such as oxidative stress, inflammatory response and renal function impairment in HUA patients with hyperlipidemia. Overall, these studies provide important clues to our understanding of the metabolic mechanisms of HUA and its associated complications; however, these studies have some limitations. First of all, the animal model can not completely simulate the physiological and pathological state of human beings, and the results need to be further verified in human samples. Secondly, metabolomics analysis requires a large number of samples, and a small number of samples will affect the reliability of the results. Finally, these studies focus on metabolic pathway changes, but do not delve into how they contribute to disease onset and progression.

### 2.2. Transcriptomics in HN mechanism research

Transcriptomics has revealed the regulation and mechanism of gene expression, as well as the genes or pathways related to HN, such as oxidative stress,^[28]^ inflammation,^[29]^ apoptosis,^[30]^ autophagy,^[31]^ and fibrosis,^[32]^ providing detection means for the discovery of molecular markers or therapeutic targets of HN. Li N et al^[33]^ used transcriptomic RNA sequencing technology to study the molecular mechanism of HN induced by HUA and found that HN affects the expression of a variety of non-coding Rnas and signaling pathways in the kidney, especially the PI3K-Akt and NF-κB signaling pathways related to inflammatory response. Through ceRNA network analysis, PTAFR, NLRP12, and ADAM19 were selected as key genes and potential biomarkers of HN, which revealed the effect of HUA on kidney gene expression. Ren et al^[34]^ found through RNA-Seq transcriptomics that natural flavonoids pectinoaglytin can regulate the expression of related genes in kidney tissue by inhibiting the signaling pathway of TGF-β1 and transcription factors SMAD3 and STAT3, and alleviate renal tubulointerstitial fibrosis in HN mice and urate-treated mouse renal epithelial cells. Hui Zhang et al^[35]^ found through transcriptomics that cadmium exposure interfered with purine metabolism, immune-mediated inflammation and energy metabolism, which was characterized by the increase of xanthine and hypoxanthine, and the significant activation of peroxisome proliferator activated receptor in the liver of mice after cadmium exposure, thus accelerating the progression of HUA. Yao Tan et al^[36]^ verified the effect of diosgenin on HUA at the gene level through transcriptomics. The results showed that the levels of 89 genes in the model group were significantly changed, and diosgenin treatment restored the levels of 6 genes, suggesting that tx1b, Tsku, Tmem163, Psmc3ip, Tcap, and Tbx15 were mainly involved in cell cycle and energy metabolism. Guo et al^[37]^ used transcriptomics to analyze the effect of alpha-glucin on HUA mice. The results showed that α-glucin could reduce UA production by decreasing XOD activity. Regulate the expression of uric acid transporter, down-regulate URAT1 and GLUT9, up-regulate ABCG2 and OAT1, and promote the excretion of UA. In addition, alpha-glucin also affected IL-17, chemokines and PI3K-AKT signaling pathway, thereby protecting kidney function in HUA mice. These studies have revealed the molecular mechanism of HUA and its induced HN through transcriptomics, providing many valuable insights. However, most studies focus on the role of a single factor or a single compound, and the occurrence and development of HUA and HN are often the result of a combination of multiple factors. Animal models or cellular models may not fully simulate the complex physiological environment and pathological processes of the human body. Therefore, when exploring treatment strategies for HUA and HN, it is necessary to consider multiple factors comprehensively and verify them in more complex physiological environments in order to find more effective treatment methods.

### 2.3. Study on the mechanism of intestinal microecology in HN

UA is the end product of purine metabolism, and about 30% of UA is excreted through the gut or further catabolized by the gut flora. Intestinal flora is the microbial community designated to plant in the human gut, including bacteria, fungi, protozoa, viruses, etc. Among them, bacteria is the most important component, 10 times that of human cells.^[38]^ Intestinal flora forms a mutually-beneficial symbiotic relationship with the host, participating in the regulation of multiple host systems such as digestion,^[39]^ absorption,^[40]^ metabolism,^[41]^ immunity,^[42]^ and neuroendocrine.^[43]^ In recent years, intestinal flora has played an important role in the occurrence and development of HN. Intestinal flora can regulate host UA homeostasis by influencing purine metabolism, UA excretion, inflammatory response, intestinal barrier function^[44]^ and other aspects. Specific microbial taxa such as Vallitalea, Christensenella and Insolitispirillum are also closely related to the occurrence of HUA.^[45]^ HUA is also an important risk factor for HN, which can lead to oxidative stress, inflammation, fibrosis and calcification of the kidney, and then damage of renal function. Therefore, regulating intestinal flora may be an effective way to prevent and treat HUA and HN. At present, some studies have explored the effects of different intestinal flora intervention strategies on HUA and HN and their mechanisms of action. Na Li et al^[46]^ found through intestinal flora analysis that CichoriumintybusL.(Asteraceae)formula could improve intestinal flora imbalance in HN rats induced by adenine and ethambutol, increase the abundance of beneficial bacteria, restore intestinal microecological balance, and thus play a protective role in the kidney. Zhao et al^[47]^ found through the analysis of intestinal flora diversity that AstragalosideIV could reduce the urea-ammonia hepatoenteral circulation by reducing the abundance of urease-producing bacteria, thus alleviating kidney injury in HN rats. Xueling Xu et al^[48]^ found through intestinal flora that curcumin changed the intestinal flora structure of HN rats, inhibiting harmful bacteria and opportunistic pathogens in the intestine, such as Escherichia - Shigella and Bacteroides, increasing the production of beneficial bacteria and short-chain fatty acids, improving the structure and function of intestinal tissue, and reducing the level of plasma endotoxin. Thereby reducing kidney damage. Lv et al^[49]^ examined the changes of intestinal flora in feces of HUA mice by 16SrRNA sequencing and found that the abundance of inflammatory bacteria in HUA mice increased, which may be caused by up-regulating TLR2/4/5 and promoting the release of IL-1β and TNF-α, leading to immune disorders and intestinal barrier dysfunction. HUA was characterized by intestinal immune dysfunction, impaired intestinal barrier and systemic inflammation. In summary, it can be determined that intestinal flora plays an important role in HUA and HN, but current studies mainly focus on the effect of intestinal flora on uric acid metabolism, and the specific molecular mechanism is still insufficient. Although it has been confirmed that specific bacterial groups are closely related to the occurrence of HUA, the molecular mechanism of how these bacterial groups specifically regulate uric acid metabolism has not been fully understood. In addition, most studies rely on animal models, which do not fully mimic uric acid metabolism and gut microecology in humans. In particular, mouse models differ from humans in terms of uric acid metabolism, which may limit the validity of the findings in clinical applications. Therefore, most studies on HUA and HN remain in the laboratory stage and lack large-scale clinical trial data, which challenges the clinical translation of research results and makes it difficult to determine the effectiveness and safety of intestinal microbiota intervention strategies in actual treatment.

## 3. The internal relationship of association omics and its integrated application in the treatment of HN mechanism of action

### 3.1. Metabolomics and transcriptomics

As a medium of gene expression, transcriptomics collects information about many differentially expressed genes and a large number of regulatory networks, and is directly related to the genome of biological samples. Metabolomics can present the physiological state of biological samples at the metabolic level, which is the basis of the complex phenotype of biological samples, reflecting the changes of cell function and phenotype. The combined application of transcriptomics and metabolomics can realize the co-expression analysis of time-sequence expression of differential genes and differential metabolites, explore the association between differential genes and differential metabolites, identify metabolic pathways, find key differentially expressed genes, and finally clarify their potential biological significance. The combined analysis of transcriptomics and metabolomics based on bioinformatics database shows strong advantages in mining key differentially expressed genes and key metabolic pathways, and can more comprehensively and systematically analyze the mechanism of action and complex molecular functions of biological samples. Liu H et al^[50]^ used UHPLC-MS/MS technology to analyze serum and urine samples from HUA patients and identify the metabolites and pathways associated with HUA. The results showed that HUA was related to a variety of metabolic pathways, such as tryptophan, caffeine, glycerol phospholipids, sphingolipids, arachidonic acid, linoleic acid, phenylalanine, etc. In addition, the mice model of HUA was induced by potassium oxate. The effects of HUA on the kidney and the relationship between metabolism and transcription were also investigated, and it was found that HUA caused autophagy and NLRP3-mediated inflammation in the kidney. By integrating these 2 techniques, we can reveal the interrelationship and mechanism of action between metabolism and genes of HUA and its complications. However, the development of HN may change over time, and different observation time points may affect gene expression and metabolite level changes. Meanwhile, human gene expression and metabolic process may be affected by multiple factors such as age, sex, lifestyle and genetic background. Further research is needed to more deeply explore the genetic and metabolic characteristics of HN and how they change over time and in different individuals.

### 3.2. Metabolomics and intestinal microecology

Metabolomics and intestinal microecology are 2 kinds of techniques that can reflect the metabolism and flora status of organisms from different levels. Zhu et al^[51]^ used 16SrDNA sequencing and metabolomics techniques to find that O.aristatus could reduce the abundance of harmful bacteria such as NK4A136 group of Rumenococcaceae and Trichomorhiaceae in intestinal tract, and at the same time increase the levels of 17 beneficial metabolites such as lactose, 4-oxo-valeric acid and butyric acid in intestinal tract. And reduce the level of 55 harmful metabolites such as flavin adenine dinucleotide. Libin Pan et al^[52]^ studied the metabolomics and microbiome characteristics of HN rats and found that amino acid metabolism in HN rats was unbalanced, conditioned pathogens increased, beneficial bacteria decreased, and nitrogen cycling was impaired. Wang et al^[53]^ found changes in metabolites and intestinal flora in HUA rats, involving 6 metabolic pathways and 2 phyla, and considered indoxyl sulfate and n-acetylglutamic acid in urine as potential biomarkers of HUA. Wang et al^[54]^ used astragalus to treat HUA mice and found that Astragalus could reduce UA level, improve urea cycle, protect intestinal barrier, inhibit TLR4/NF-κB signaling pathway, and alleviate kidney inflammation by regulating intestinal flora and metabolism, thus alleviating symptoms of HUA, which was consistent with Deng S.^[55]^ It is believed that Astragalus protects mice from HUA damage through the microbiome metabolism axis. Halimulati et al^[56]^ found through non-targeted urine metabolomics analysis and metagomic sequencing of intestinal flora that amanita can regulate the balance of liver and kidney function, renal uric acid transporters, intestinal microbes and metabolism, kidney injury-related metabolites and purine metabolism, as well as the mechanism of entero-renal axis, effectively reducing UA levels in HUA rats. Although the above study found an association between HUA and gut microbiota and metabolites, it was not clear whether this association was causal or merely correlated. For example, does HUA cause changes in intestinal flora and differences in metabolites, or do changes in intestinal flora and differences in metabolites cause HUA, or are there common causes between the two? In other words, in the context of HUA and HN, is it the metabolite level that changes first, or the intestinal flora that changes first, or the change of metabolite level that affects the homeostasis of the intestinal flora? These are all issues that need further study in the future.

### 3.3. Transcriptomics and intestinal microecology

Intestinal microecology plays an important role in maintaining host health and homeostasis. The rise of metagenomics in the 20th century has largely solved the problem that most microorganisms are difficult to study because they cannot be isolated and cultured, avoiding the experimental biases that are prone to occur in the application of other inherent techniques (such as polymerase chain reaction), and also eliminating some time-consuming and labor-consuming experimental steps (such as library construction). However, this technique is still unable to determine the structure and metabolism of microbial communities, especially the response of microorganisms to their adaptation to the environment. Transcriptomics can link the biological communities and their functions under specific conditions, and conduct various related functions of the whole population. It can directly detect the transcriptional active genes, focus on the active communities in the sample, and reduce the complexity of the community. This method can describe the metabolically active components in the microbial community and provide a new method for the identification and analysis of key functional genes of specific varieties of the microbial community. He et al^[57]^ found an insulating site in the Escherichia coli genome, inserted the uricase expression box, and created an engineered strain of EcNC6. EcNC6 can efficiently express and transport uricase, colonize near small intestinal epithelial cells, utilize UA and oxygen in blood, reduce UA levels, relieve the inflammation of kidney, beta cells and intestinal flora related to HUA, and regulate the metabolism of arginine and ornithine. Guo et al^[58]^ studied the effects of HUA on intestinal barrier function and intestinal permeability. They used transcription-activator-like effect nuclease technology to knockout Uox gene in a mouse model, measured the levels of UA, TNF-α, IL-6, and uremic toxin in serum and intestinal tissue, and observed the pathological changes in the intestine. They found that Uox-KO mice had reduced intestinal barrier function, sparse intestinal villi, mucosal and submucosal edema, and increased intestinal permeability. These results suggest that HUA may disrupt the integrity of the intestinal epithelium, leading to intestinal damage. This will lead us to ponder whether it is possible to detect significantly expressed proteins in the gut through transcriptomics and analyze the changes in the gut microbiota after HN by analyzing the diversity of the gut microbiota, so as to treat HN by targeting proteins that intervene in the gut and kidney.

## 4. Discussion

HN is a metabolic disease of kidney injury caused by elevated serum UA level due to purine metabolism disorder.^[59]^ HN is a common global health problem with increasing incidence and prevalence, especially in developing countries.^[60]^ In addition to kidney damage, HN patients are at risk for complications such as gout, kidney stones, and cardiovascular disease. HN seriously affects the quality of life and prognosis of patients, and increases the consumption of medical resources and the burden of social economy. The occurrence and development of HN are influenced by many factors, among which genetics,^[61]^ diet,^[29]^ drugs,^[62]^ and endocrine^[63]^ all play a role. In recent years, some basic and clinical studies have also revealed some gene variants, signaling pathways,^[64]^ and cytokines related to HN.^[65]^ However, these factors cannot fully explain the pathogenesis and individual differences of HN, and there are still some problems such as small sample size, poor reproducibility, complicated data analysis, and difficult interpretation of results. Therefore, it is necessary to explore the pathogenic factors and molecular markers of HN from a deeper and broader perspective, in order to improve the diagnostic accuracy of HN, predict the risk, guide the treatment plan, and evaluate the therapeutic effect.

In HN research, the role of metabolomics, transcriptomics and intestinal flora has become increasingly apparent. Metabolomics technology can detect the levels of purine, pyrimidine, taurine and other metabolites, so as to analyze the abnormal metabolic pathway of UA. Transcriptomic analysis showed that HN patients had abnormalities in purine metabolism, oxidative stress and energy metabolism. Intestinal microecology can regulate serum UA levels in a variety of ways, thus affecting the occurrence and development of HN. First, there are anaerobic bacteria in the gut with UA-degrading enzymes that can convert UA into 5-hydroxy-isUA, which can then be further broken down into CO2 and ammonia, thereby reducing serum UA levels. Secondly, metabolites of intestinal flora can affect renal excretion of UA through different signaling pathways, among which short-chain fatty acids and bile acids are most closely related to HN. Finally, the imbalance of intestinal flora can lead to increased intestinal permeability, which allows endotoxins and other inflammatory factors to enter the bloodstream and aggravate the progression of HN. These harmful bacteria can disrupt the intestinal barrier, release endotoxins and pro-inflammatory cytokines, and inhibit the excretion of UA by the kidneys, resulting in increased serum UA levels.

Metabolomics, transcriptomics and intestinal microecology are important means to study HN, which can reveal the characteristics and mechanism of HN from different levels and perspectives. However, a single technique often cannot fully explain the complexity and heterogeneity of HN, so integrated analysis is needed to improve the reliability and interpretation of the data and discover new biomarkers and therapeutic targets. However, multiple omics techniques also have some limitations, such as data quality, size, type, source and interpretation. To overcome the limitations of multi-omics techniques, we need to improve data quality, reduce data size, process data types, consider data sources, and interpret data results properly. In the future, with the development and innovation of technology, multi-omics technology will play a greater role in HN research. Finally, for future research direction, we propose the following hypothesis, although the essence of HN is the deposition of urate in the kidney, the normal operation of the body often depends on the communication between organs. For example, exercise interventions can protect the heart by the contraction/relaxation of skeletal muscles communicating with the heart, and drug interventions can protect target organs by the liver/kidneys communicating with other organs. We know that the production of UA is mainly carried out in the liver, and the excretion of UA requires the participation of the intestine. So, can we sequence RNA in the liver and gut in the context of HN and compare differentially expressed genes between different groups using Venn diagrams? We can identify up-regulated/down-regulated genes to find genes that encode secreted proteins. In doing so, we can also make comparisons with other organs to determine whether the target protein in the liver/gut is secreted only in the liver/gut, and whether the change in the target protein level is a protective response of the liver/gut to HN, thereby enabling potential gut-liver/kidney communication. If the above hypothesis holds, when we find the target protein, we can knock it out to determine whether the loss of the target protein in hepatocytes/target protein in intestinal epithelial cells improves kidney function and kidney pathology in HN mice. If there is a significant improvement, then we can explore the upstream and downstream mechanisms of the protective effect of knocking out target proteins on HN to determine whether the body can protect damaged organs and restore their normal function through interorgan communication. Of course, all of this requires the involvement of multiple omics techniques.

In summary, our multi-omics comprehensive analysis revealed the potential pathogenesis of HN, which mainly includes the following aspects: disturbance of amino acid metabolism leads to increased UA production; energy metabolism inhibition leads to mitochondrial dysfunction; increased nucleotide metabolism resulted in decreased UA excretion; lipid metabolism disorder and bile acid metabolism imbalance lead to intestinal barrier damage; ROS level is unbalanced due to disordered REDOX process; Cell cycle and apoptosis imbalance leading to cell damage and death; enhanced signal transduction and inflammatory response lead to abnormal immune response; and the imbalance of intestinal flora leads to changes in the microecological environment.

## 5. Conclusion

In this paper, the mechanism of HN was discussed from the perspectives of metabolomics, transcriptomics and intestinal microecology. The results show that multi-omics techniques can reflect the characteristics of HN, such as UA metabolic abnormalities, oxidative stress response, inflammatory response, intestinal flora imbalance, etc. These characteristics may interact and influence each other, and jointly participate in the occurrence and development of HN.

## Author contributions

**Conceptualization:** Kai qing Li, Xue Xia, Ying Tong.

**Data curation:** Tong Fu, Yanchun Ma, Yingwei Wang, Mingming Fan, Songyan Wang.

**Supervision:** Guoli Xing.

**Writing – original draft:** Kai qing Li.

**Writing – review & editing:** Kai qing Li, Guoli Xing, Ying Tong.
